# Integration of advanced 3D SPECT modelling for pinhole collimators into the open-source STIR framework

**DOI:** 10.3389/fnume.2023.1134774

**Published:** 2023-04-18

**Authors:** Matthew Strugari, Carles Falcon, Kjell Erlandsson, Brian F. Hutton, Kimberly Brewer, Kris Thielemans

**Affiliations:** ^1^ Biomedical MRI Research Laboratory, IWK Health Centre, Halifax, NS, Canada; ^2^ Department of Physics and Atmospheric Science, Dalhousie University, Halifax, NS, Canada; ^3^ Neuroimaging Group, Barcelonaßeta Brain Research Center, Barcelona, Spain; ^4^ Institute of Nuclear Medicine, University College London, London, United Kingdom; ^5^ Department of Diagnostic Radiology, Dalhousie University, Halifax, NS, Canada; ^6^ Centre for Medical Image Computing, University College London, London, United Kingdom

**Keywords:** Image reconstruction, molecular imaging, Monte Carlo methods, nuclear medicine, open-source software, SPECT

## Abstract

Single-photon emission computed tomography (SPECT) systems with pinhole collimators are becoming increasingly important in clinical and preclinical nuclear medicine investigations as they can provide a superior resolution-sensitivity trade-off compared to conventional parallel-hole and fanbeam collimators. Previously, open-source software did not exist for reconstructing tomographic images from pinhole-SPECT datasets. A 3D SPECT system matrix modelling library specific for pinhole collimators has recently been integrated into STIR, an open-source software package for tomographic image reconstruction. The pinhole-SPECT library enables corrections for attenuation and the spatially variant collimator–detector response by incorporating their effects into the system matrix. Attenuation correction can be calculated with a simple single line of response or a full model. The spatially variant collimator–detector response can be modelled with a point spread function and depth of interaction corrections for increased system matrix accuracy. In addition, improvements to computational speed and memory requirements can be made with image masking. This work demonstrates the flexibility and accuracy of STIR’s support for pinhole-SPECT datasets using measured and simulated single-pinhole SPECT data from which reconstructed images were analysed quantitatively and qualitatively. The extension of the open-source STIR project with advanced pinhole-SPECT modelling will enable the research community to study the impact of pinhole collimators in several SPECT imaging scenarios and with different scanners.

## 1. Introduction

Single-photon emission computed tomography (SPECT) is based on the detection of individual γ-rays emitted from a radiotracer distribution within a subject. An Anger camera detects the γ-rays with a scintillation crystal and associated electronics after passing through a collimator (1). The collimator aperture permits the passage of γ-rays from specific directions, and the pattern of photon interactions in the scintillation crystal forms a 2D projection image of the tracer distribution in the subject. A series of projection images acquired from different angles can be subsequently used to reconstruct the 3D radiotracer distribution in a tomographic image.

The design of the collimator in terms of hole size, material, and overall geometry, among other factors, affects the spatial resolution and sensitivity of a SPECT system. Several designs exist, including but not limited to parallel-hole, slanthole, converging and diverging, fanbeam, and pinhole collimators (2). Therefore, the choice of collimator design is application-dependent for channelling photons of different energies, magnifying or minifying images, or selecting between image quality and imaging speed. Although parallel-hole and fanbeam collimators are conventionally used when imaging small fields-of-view (FOVs), pinhole collimators can provide a superior resolution-sensitivity trade-off (3). In addition to the successful application of pinhole SPECT systems in small animal imaging, there has been a resurgence in the use of pinhole collimators for clinical cardiac and brain studies and when imaging small FOVs (4).

While pinhole SPECT has regained popularity in clinical and preclinical investigations of molecular imaging agents, no open-source software solutions are available for reconstructing pinhole SPECT datasets. However, recent efforts have led to the integration of a 3D SPECT system matrix modelling library for pinhole collimators into the open-source Software for Tomographic Image Reconstruction (STIR). The STIR package is an object-oriented library implemented in C++ that provides a framework for research in the processing and reconstruction of emission tomography studies (5). Initially written to support positron emission tomography (PET) data, STIR was previously extended to handle SPECT data with parallel- and converging-hole collimators (6,7). This was achieved by integrating parts of the SPECT Reconstruction Library (developed at the University of Barcelona) into STIR (8–11). The expansion of STIR’s support for pinhole collimators marks the first open-source platform for reconstructing pinhole SPECT datasets, which is important for advancing molecular imaging techniques and technologies.

This work aims to demonstrate the capabilities of STIR’s support for pinhole SPECT datasets. The pinhole code uses a similar implementation strategy as the previously integrated SPECT collimator modelling. The library enables corrections for attenuation and the spatially variant collimator–detector response by incorporating their effects into the system matrix.

## 2. Technical description

Similar to the original SPECTUB implementation, the new pinhole SPECT implementation is referred to as PinholeSPECTUB and includes a dedicated reader for pinhole SPECT projection data in Interfile format (12), with some adaptations as pinhole collimators are not supported in Interfile. The pinhole SPECT Interfile reader utilises the projection matrix size, pixel scaling factor, and detector radius defined at the face of the scintillation crystal. System matrix calculation is executed with the ProjMatrixByBinPinholeSPECTUB projector class derived from the existing STIR ProjMatrixByBin class, and detector and collimator parameter files are utilised in addition to the usual STIR parameter file. The parameter files are text files that use an Interfile-like syntax. They are composed of keywords corresponding to the names of the various reconstructions and matrix parameters with the values entered next to them. Sample parameter files for configuring the PinholeSPECTUB projector can be found in the Appendix, and a detailed description of all parameters can be found in STIR’s documentation.

**Figure 1:**
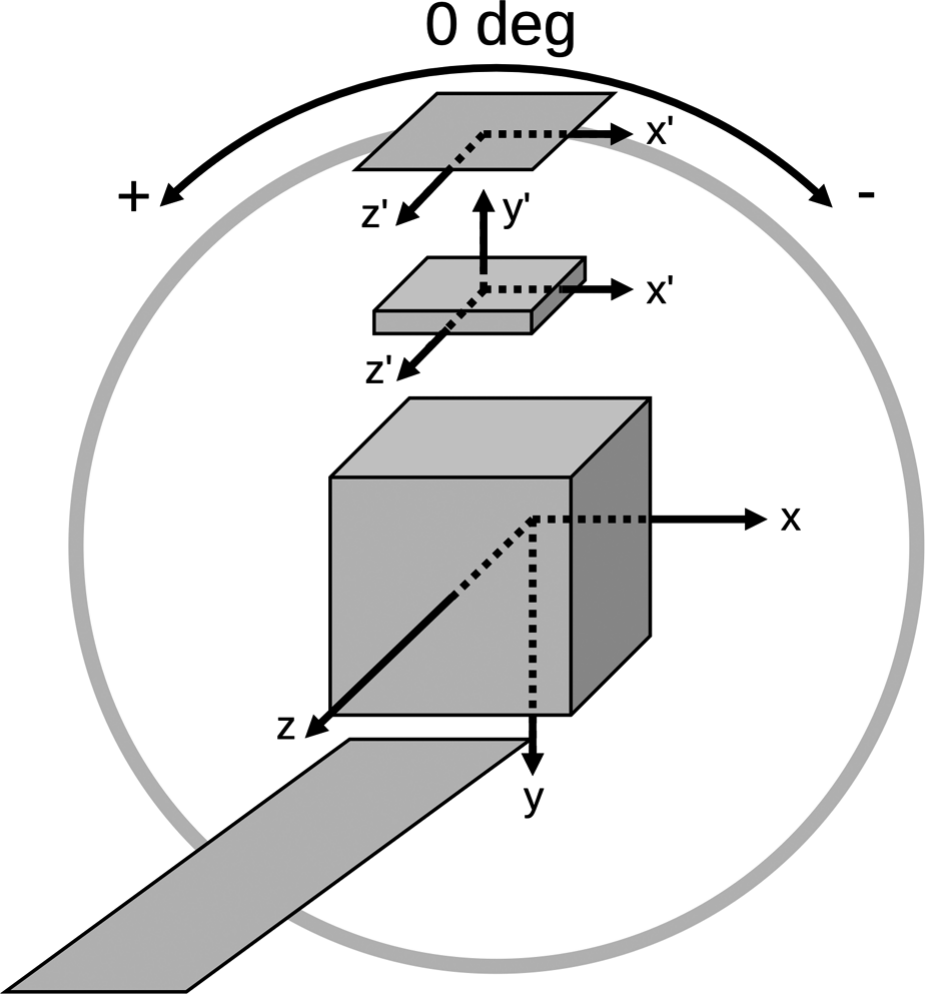
PinholeSPECTUB system of reference and sign criteria illustrated for a polygonal collimator setup. Note that the projection matrix adheres to STIR’s coordinate system as indicated by the x, y, and z axes. The detector and collimator use a rotating frame of reference where the transaxial $x^{\prime }$ and axial $z^{\prime }$ axes coincide with STIR’s axes when the detector is at $0^{\circ }$. The collimator uses a right-handed coordinate system as indicated by the $y^{\prime }$ axis, which points toward the detector. Further information is given in the text and STIR’s documentation.

The detector file defines the intrinsic resolution for point spread function (PSF) correction, scintillation crystal attributes for depth of interaction (DOI) correction, and orbit information for the acquisition (i.e., number of orbits, number of angles, initial angle, angular increment—positive for counterclockwise and negative for clockwise rotation, and axial position with respect to the reconstructed volume). Note that only circular camera orbits are supported at this time. The collimator file defines the radius of rotation and geometry for cylindrical or polygonal collimators (i.e., the detector element exposed by the pinhole, hole position, shape—rectangular or round, size, tilt, and acceptance angle). An illustration of the pinhole SPECT system matrix geometry for a polygonal collimator setup is shown in Figure 1.

The system matrix weights the contribution of each image voxel along the line of response (LOR) to each detector element. Corrections can be made for increased system matrix accuracy by modelling the effects of intrinsic PSF, DOI, and attenuation (ATT) when configuring the STIR parameter file. When PSF correction is disabled, a geometrical approach is applied by considering the projection of the pinhole on the detector. This provides higher computational speed and a reduced memory requirement compared to the PSF approach, but is less accurate. When PSF correction is enabled, the projection of the hole is convolved with the PSF in detector space to account for the blurring effects of the camera. Values parsed from the parameter file define the number of standard deviations to be considered in the PSF, along with the subsampling factor to temporarily reduce PSF resolution for increased calculation accuracy, before downsampling the final PSF to the bin size. Furthermore, when PSF or DOI corrections are enabled, an additional parsed parameter sets the spatial sampling interval for PSF and DOI distributions.

Enabling DOI correction subdivides the scintillation crystal using Bresenham’s line algorithm (13) to calculate the crystal attenuation and DOI along the LOR. If DOI correction is disabled, half the crystal thickness is added to the detector radius. When attenuation correction is enabled, a simple correction can be applied where the same attenuation factor is applied for the whole PSF, or a full correction can be performed where different attenuation factors are applied for each bin of the PSF (7). Further improvements to speed and memory can be made with image masking using the default cylinder, an attenuation map, or a mask file. The default cylinder is based on the object radius in the image volume. It is essential to set the object radius greater than or equal to the size of the object in the attenuation map or mask file when masking, as the matrix weights are calculated according to this value. Failure to do so will result in an error. The projection matrix can be kept in memory or calculated per projection angle. In the latter case, the memory is released before starting calculations on a new angle, reducing memory requirements but increasing computation time for iterative reconstruction algorithms.

## 3. Materials and methods

To test the pinhole-SPECT implementation in STIR, the Spark silicon-photomultiplier (SiPM)-based preclinical SPECT system was used with a single-pinhole (SPH) collimator (Cubresa Inc., Winnipeg, Canada). Previous work characterised the system with the National Electrical Manufacturers Association (NEMA) NU 1-2018 Standards for Performance Measurements of Gamma Cameras, and a corresponding Geant4 Application for Tomographic Emission (GATE) Monte Carlo model was validated (14). Excellent agreement was found between measurement and simulation, with differences on the order of a few percent, supporting the accuracy and detailed analysis of simulated data in this study.

The Spark has a fixed rotation range of 270^∘^ from a starting angle of 180^∘^. Angular increments of 3^∘^ were used for data acquisition based on NEMA’s specification (15). GATE simulation results (16) were output to Rapid Object-Oriented Technology (ROOT) format (17) and converted to Cubresa’s list mode format. Projection data with 0.5 mm bins were generated from measured and simulated list mode data using a 30%-wide energy window centered at 140 keV. Projection images were then converted from Cubresa’s format to Interfile format for use with STIR. Parameter files were configured as necessary with a full attenuation correction model, a PSF subsampling factor of 1, a maximum number of PSF standard deviations of 2, and a spatial resolution of 0.1 mm when sampling distributions in PSF or DOI corrections. Unless explicitly stated, images were reconstructed in the entire FOV using an object radius of r=23.0 mm.

Simulations and image reconstructions were performed on an HP Z820 workstation operating Ubuntu 18.04.5 LTS with two Intel Xeon E5-2630 2.3 GHz hexa-core CPUs and 64 GB of 1600 MHz DDR3 memory. The SPH-SPECT data for quantitative image assessment were simulated with GATE v9.0, while qualitative image assessment used *in vivo* data. Tomographic images were reconstructed with STIR v5.1.0 on a single CPU core as the PinholeSPECTUB projector class has not yet been configured to use the OpenMP or Message Passing Interface capabilities of STIR, which would allow several computations to be performed in parallel. Note that pre-corrected projection data is expected to be input into the projection matrix. Therefore, measured data were corrected with energy, linearity, and uniformity calibrations, while simulated data required no calibration.

### 3.1. Quantitative assessment of reconstructed data

#### 3.1.1. Phantom simulations and data generation

Phantom data were simulated with three different subjects containing technetium-99m (^99*m*^Tc): a NEMA Micro-PET IQ phantom, a mouse-sized NEMA triple line source scatter phantom, and a volumetric cylinder. The IQ phantom (outer diameter ø_OD_ = 33.5 mm, length L=63.0 mm) was made from polymethyl methacrylate containing three different sections: a spillover section with water and air, a uniform section (inner diameter ø_ID_ = 30.0 mm, L=15.0 mm), and a section with five hot rods (ø_ID_ ={1,2,3,4,5} mm, L=20.0 mm). The triple line source scatter phantom (ø_OD_ = 25.4 mm, L=60.0 mm) was made from acrylic to house three precision glass capillary tubes (ø_OD_ = 0.8 mm, ø_ID_ = 0.4 mm), with one located at the center and two with a 10.0 mm radial offset separated by 90^∘^. The volumetric cylinder (ø_OD_ = 28.0 mm, L=55.0 mm) was made from acrylic with a uniform section of radioactivity (ø_ID_ = 26.0 mm, L=21.0 mm). Attenuation maps were produced with GATE to delineate regions of interest (ROIs) and correct for attenuation in the triple-line source phantom and volumetric cylinder.

Table 1 summarises the simulated phantom acquisitions, projection and reconstruction matrices, reconstruction algorithms, and applied analyses which are further described in the proceeding subsections. Iterative reconstruction algorithms and matrix corrections were used to assess figures of merit in terms of computation cost, contrast-to-noise ratio, resolution, uniformity, and variability.

**Table 1:** Summary of simulated ^99*m*^Tc phantom acquisitions and reconstructions.

Subject	Activity	Acquisition	Projections	Projection matrix	Reconstruction matrix	Algorithm	Analysis
IQ phantom	50 MBq	Forward proj.	120 (8 subsets)	90×90 px, 1.0 mm	120×92×92 vx, 0.5 mm	OSEM	Computation cost
IQ phantom	50 MBq	3600 s	91 (7 subsets)	208×208 px, 0.5 mm	230×184×184 vx, 0.25 mm	OSEM, OS-OSL-MRP, OS-SPS-QP	Hot rod CNR
Line source	30 MBq	5460 s	91 (7 subsets)	208×208 px, 0.5 mm	230×184×184 vx, 0.25 mm	OSEM	Resolution
Cylinder	20 MBq	910 s	91 (7 subsets)	208×208 px, 0.5 mm	230×184×184 vx, 0.25 mm	OSEM	Uniformity & CV

OSEM, ordered subsets expectation maximisation; OS-OSL-MRP, ordered subsets one step late with median root prior (penalisation factor, PF=1.0); OS-SPS-QP, ordered subsets separable paraboloidal surrogate with quadratic prior (PF=0.3).

CNR, contrast-to-noise ratio; CV, coefficient of variation.

#### 3.1.2. Computation cost with different matrix corrections

To compare computation costs for different types of matrix corrections, a forward projection of the IQ phantom was made with 120 views over 360^∘^ using a reduced matrix size (see Table 1). Images with different matrix configurations were reconstructed with the ordered subset expectation maximisation (OSEM) algorithm (18) using eight subsets and 40 subiterations. Matrices were configured for no corrections (N-C), attenuation correction (ATT-C), DOI correction (DOI-C), PSF correction (PSF-C), all corrections (PSFATTDOI-C), and all corrections with masking (PSFATTDOIM-C) using the default cylindrical mask (r=17.0 mm). Maximum RAM and CPU time were recorded with Ubuntu’s /usr/bin/time -v command when calling STIR’s OSMAPOSL program from the command line. Memory and CPU time requirements were compared between storing the matrix in memory and calculating it per projection angle.

#### 3.1.3. Contrast-to-noise ratios in the IQ phantom

**Figure 2:**
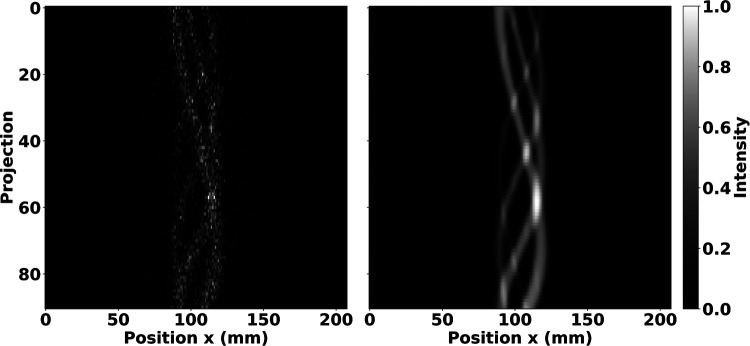
Projection of the IQ phantom hot rod region displayed in a 2D sinogram arrangement showing the GATE simulated data (**left**) and the STIR forward projection of the radioactive source distribution adding attenuation, DOI, and PSF degradation (**right**). Sinograms were normalised by the maximum pixel count. The sinograms show good agreement despite the relatively low count statistics associated with the SPH-SPECT simulation.

Sample sinograms of the IQ phantom hot rods are shown in Figure 2 from the GATE simulation and the STIR forward projection, including attenuation, DOI, and PSF effects. Despite the relatively low count statistics associated with the SPH-SPECT simulation, the visual agreement between these sinograms supports that the implementation of the PinholeSPECTUB projector matrix in STIR is suitable for pinhole SPECT datasets.

To compare different reconstruction algorithms, the contrast-to-noise ratio (CNR) for each hot rod i in the IQ phantom was assessed using

(1) $${\mathrm{CNR}}_{i}=\frac{\left|I_{i}-I_{r}|/(I_{i}+I_{r}\right)}{\sigma /\mu }.$$

Here, $I_{i}$ is the mean intensity of the ith hot rod delineated by the attenuation map, $I_{r}$ is the mean intensity of the reference ROI central to the hot rods (ø=5.4 mm, L=15.0 mm), and σ and μ are the standard deviation and mean intensity, respectively, in an ROI central to the uniform volume (ø=18.0 mm, L=11.25 mm). To elaborate, the cylindrical ROIs covered 60% of the active diameter and 75% of the active length based on NEMA’s methodology, except for hot rod ROIs, which used the entire diameter and length in analysis. Note that the coefficient of variation CV is expressed in the denominator of Eq. (1):

(2) $$\mathrm{CV}=\frac{\sigma }{\mu }.$$

The reconstruction algorithms chosen for CNR comparisons were OSEM, ordered subsets one step late with median root prior (OS-OSL-MRP) using a penalisation factor of PF=1.0 (19), and ordered subsets separable paraboloidal surrogate with quadratic prior (OS-SPS-QP) using PF=0.3 and relaxation parameters of α=1.0 and γ=0.1 (20). The OS-SPS-QP algorithm was initialised with the OSEM image after 21 subiterations. Hot rod CNR was calculated for each algorithm and plotted over the number of subiterations.

#### 3.1.4. Resolution in the scatter phantom

To compare resolution with different types of corrections available in the PinholeSPECTUB projector, the triple line source scatter phantom was reconstructed with the OSEM algorithm in the following configurations: N-C, ATT-C, DOI-C, PSF-C, and PSFATTDOI-C. The in-plane resolution was calculated according to NEMA’s methodology from the average full width at half maximum (FWHM) in x and y directions in three 3.5-mm-thick transverse slices: one at the center and two at ±14.5 mm. The average of all x and y FWHM results was calculated for each matrix configuration and plotted over the number of subiterations.

#### 3.1.5. Uniformity and variability in the volumetric cylinder

To compare uniformity and variability with different types of corrections available in the PinholeSPECTUB projector, the volumetric cylinder was reconstructed with the OSEM algorithm in the following configurations: N-C, ATT-C, DOI-C, PSF-C, and PSFATTDOI-C. Variability was assessed from the coefficient of variation using Eq. (2), and uniformity U was calculated as

(3) $$U=\frac{I_{\max}-I_{\min}}{I_{\max}+I_{\min}}$$

where $I_{\max}$ and $I_{\min}$ refer to the maximum and minimum intensities in the ROI central to the uniform volume (ø=15.6 mm, L=15.75 mm). Smaller values of uniformity and variability correspond to better image quality. The uniformity and variability results were separately plotted over the number of subiterations for each matrix configuration.

### 3.2. Qualitative assessment of reconstructed *in vivo* data

A previously acquired *in vivo* dataset was chosen to demonstrate qualitative image results from an investigation of novel radiotracers for Alzheimer’s disease diagnosis (21, 22). As summarised in Table 2 and (23), a B6SJLF1/J mouse was administered an intravenous tail-vein injection with a 28 MBq iodine-123 (^123^I)-labelled cholinesterase agent. The SPH-SPECT acquisition commenced 2 h post-injection, and the acquired data were reconstructed with the maximum likelihood expectation maximisation MLEM algorithm in nine iterations (24). A subsequent micro-CT (μμCT) scan was acquired with a Triumph LabPET4/CT (TriFoil Imaging, Chatsworth, USA) using an X-ray tube potential of 70 kVp and exposure of 17.8 mAs over 512 projections. The μμCT image was reconstructed with filtered back-projection (FBP) and a ramp filter in a 512×512×512 matrix having 0.1 mm isotropic voxels. The fused SPECT/CT image was visually inspected for uptake in different organs and any notable features.

**Table 2:** Summary of *in vivo* ^123^I acquisition and reconstruction.

Subject	Activity	Acquisition	Projections	Projection matrix	Reconstruction matrix	Algorithm	Analysis
*In vivo* mouse	28 MBq	3600 s	91 (1 subset)	208×208 px, 0.5 mm	230×184×184 vx, 0.25 mm	MLEM	Qualitative review

## 4. Results

### 4.1. Quantitative assessment of reconstructed data

**Figure 3:**
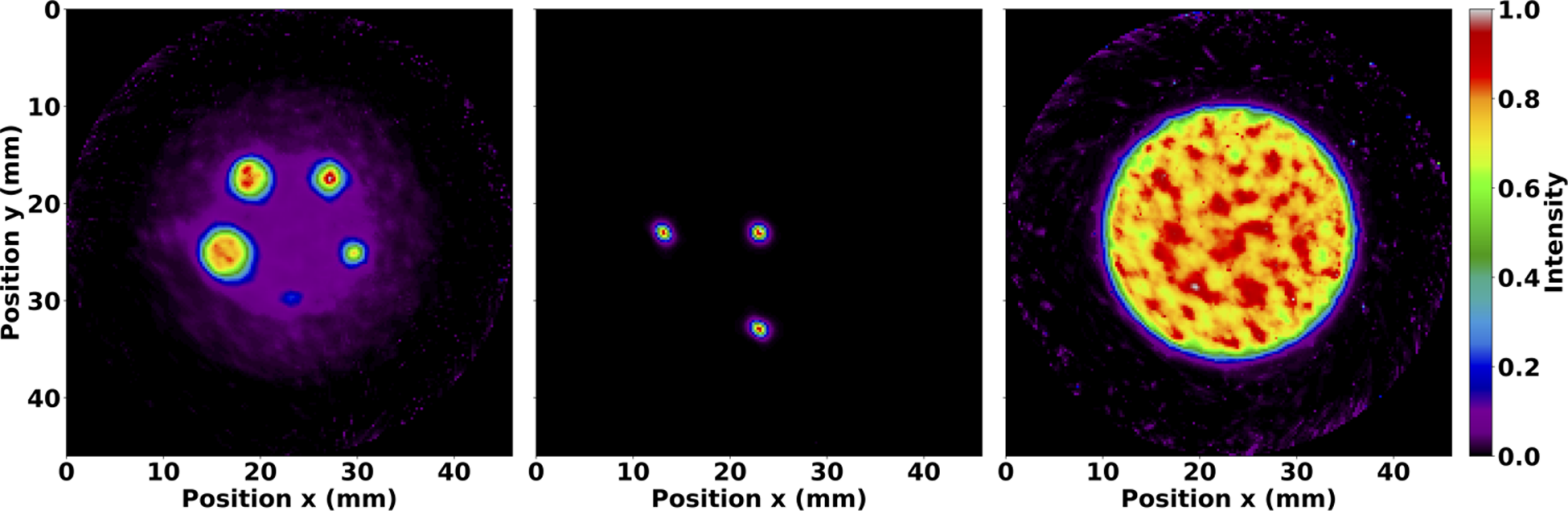
Normalised axial sum of OSEM images after 35 subiterations with seven subsets and no matrix corrections. Images are shown for the IQ phantom hot rods (**left**), mouse-sized NEMA line source phantom (**middle**), and volumetric cylinder (**right**). The IQ phantom image was summed over the length of the hot rods, whereas the other images were summed over the entire length of the reconstructed image. Note the expected distributions of ^99*m*^Tc.

Axial sums of OSEM reconstructed images from phantom simulations are shown without matrix corrections in Figure 3. These images illustrate the radioactive ^99*m*^Tc source distributions analysed in the proceeding subsections. Furthermore, they demonstrate appreciable image quality characteristics with source distributions true to their physical geometry.

#### 4.1.1. Computation cost with different matrix corrections

Table 3 summarises the time and memory requirements for OSEM reconstruction of SPH-SPECT data with different matrix corrections while keeping the matrix in memory or (re)calculating it for every projection angle. As expected, storing the matrix in memory required more memory but less CPU time than calculating it per projection angle. Comparing calculations where matrix corrections were applied independently, PSF correction required the greatest memory and the least computation time. In contrast, attenuation correction required no additional memory, and DOI correction required the greatest computation time. The combined usage of DOI and PSF corrections required even greater memory and time due to PSF correction applied at different depths in the crystal, while the inclusion of attenuation modelling further increased CPU time.

**Table 3:** Computation cost in SPH-SPECT OSEM reconstruction with 120 projections, eight subsets, and 40 subiterations.

Correction type	Matrix in memory		Matrix per projection	
	Max RAM (MB)	CPU time (s)	Max RAM (MB)	CPU time (s)
N-C	8,344	114	175	310
ATT-C	8,353	414	184	2,154
DOI-C	14,624	1,236	228	6,610
PSF-C	22,388	265	304	783
PSFATTDOI-C	31,689	2,677	380	16,421
PSFATTDOIM-C	18,368	1,495	267	8,211

N-C, no corrections; ATT-C, attenuation correction; DOI-C, DOI correction; PSF-C, PSF correction; PSFATTDOI-C, all corrections; and PSFATTDOIM-C, all corrections with masking using the default cylindrical mask (r=17.0 mm).

#### 4.1.2. Contrast-to-noise ratios in the IQ phantom

**Figure 4:**
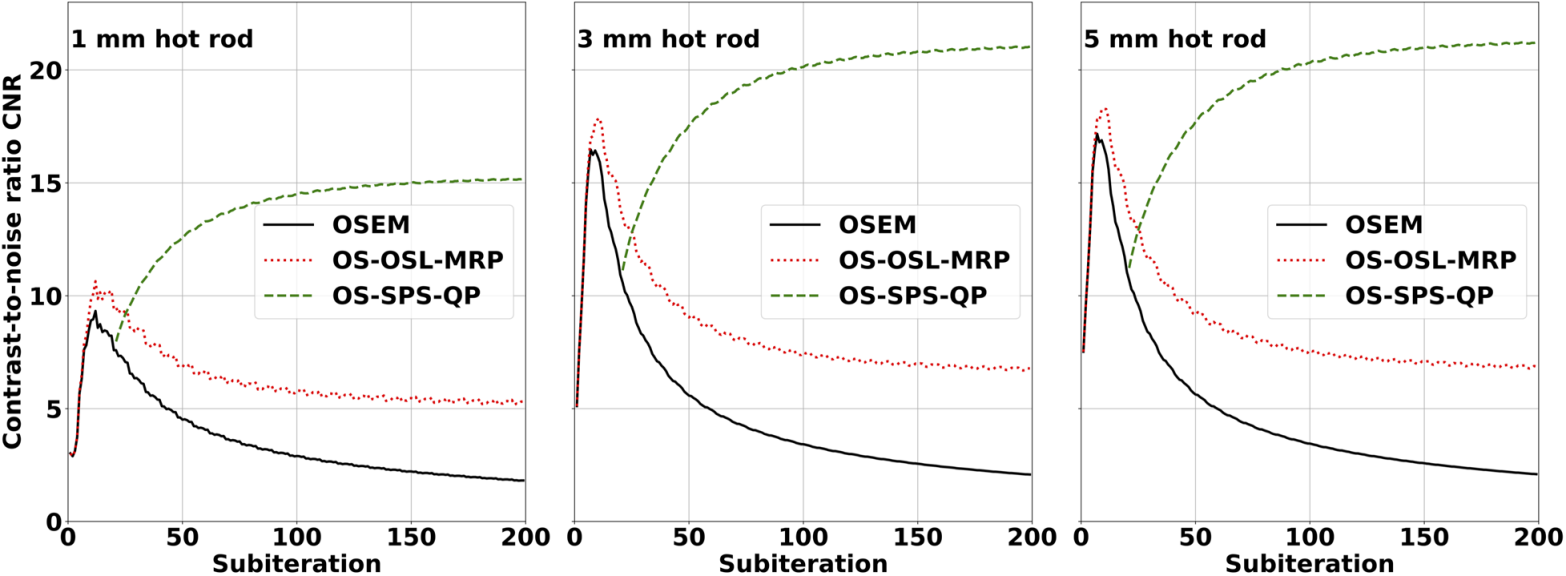
SPECT IQ phantom hot rod CNR plots for the 1 mm (**left**), 3 mm (**middle**), and 5 mm (**right**) hot rods. Images were reconstructed using OSEM (solid line), OS-OSL with median root prior (dotted line), and OS-SPS with quadratic prior (dashed line). All images were reconstructed with seven subsets and no matrix corrections, and the OS-SPS-QP reconstruction was initialised with the OSEM image after 21 subiterations. Hot rod contrast was calculated relative to the central inter-rod region void of ^99*m*^Tc, and CV was calculated in the uniform ^99*m*^Tc region. The OS-SPS-QP algorithm preserved spatial detail and effectively reduced noise while converging to a stable value.

Image quality was assessed from hot rod CNR in the IQ phantom for different reconstruction algorithms available in STIR, including OSEM, OS-OSL-MRP, and OS-SPS-QP. Figure 4 presents the performance of these algorithms based on plots of hot rod CNR over 200 subiterations. In OSEM reconstruction, the CNR reached a maximum following one complete iteration and then continually decreased with increasing subiterations due to an amplification of the variability in the uniform ROI. In OS-OSL-MRP and OS-SPS-QP reconstructions, the CNR converged to a stable value while preserving spatial detail. However, OS-OSL-MRP reached a maximum CNR following one complete iteration and then decreased toward a stable value with increasing subiterations, and OS-SPS-QP converged toward a maximum and stable value with increasing subiterations. The increase in CNR for the OS-SPS-QP algorithm can be attributed to its effectiveness in noise reduction, particularly in the uniform ROI.

#### 4.1.3. Resolution in the scatter phantom

**Figure 5:**
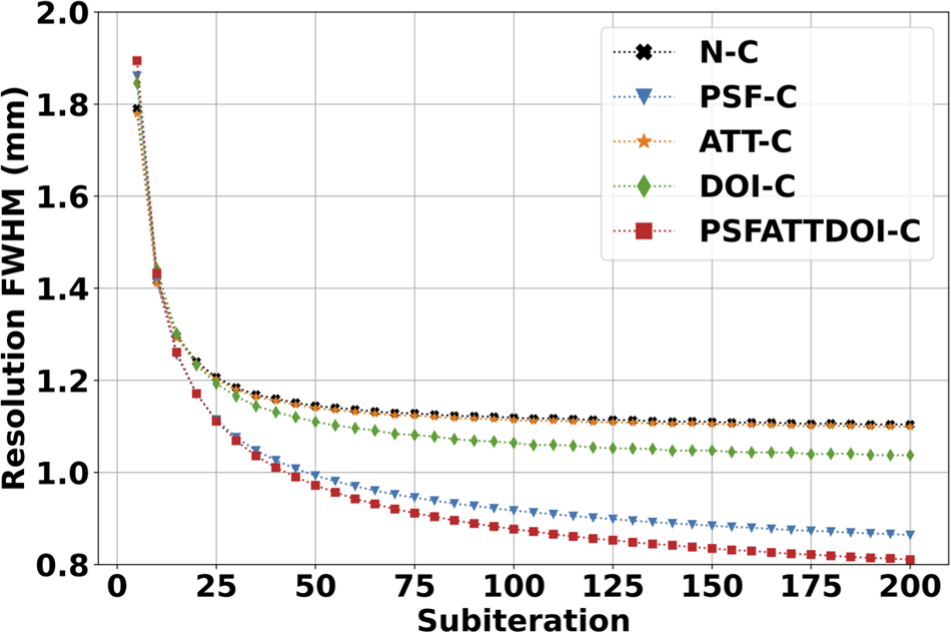
SPECT spatial resolution with scatter in the mouse-sized NEMA triple line source scatter phantom. Images were reconstructed using the OSEM algorithm with seven subsets and various matrix corrections. Resolution was calculated from the average x and y FWHM in three 3.5 mm-thick transverse slices. As expected, resolution improved with increasing subiterations in OSEM reconstruction. PSF correction provided the greatest resolution improvement compared to other independently applied matrix corrections.

Figure 5 shows a plot of the average in-plane resolution of precision line sources in the mouse-sized NEMA triple line source scatter phantom reconstructed with the OSEM algorithm. In all cases, the resolution improves as the number of subiterations increases. When comparing resolution with and without matrix corrections by averaging the FWHM across all 200 subiterations, it can be seen that attenuation correction resulted in a negligible 0.4% improvement to resolution due to its present application in a preclinical setting where attenuation effects were minimal. DOI correction provided a 4% improvement in resolution, and PSF correction provided a 16% improvement in resolution. Combining matrix corrections yielded the greatest 19% improvement in resolution.

#### 4.1.4. Uniformity and variability in the volumetric cylinder

**Figure 6:**
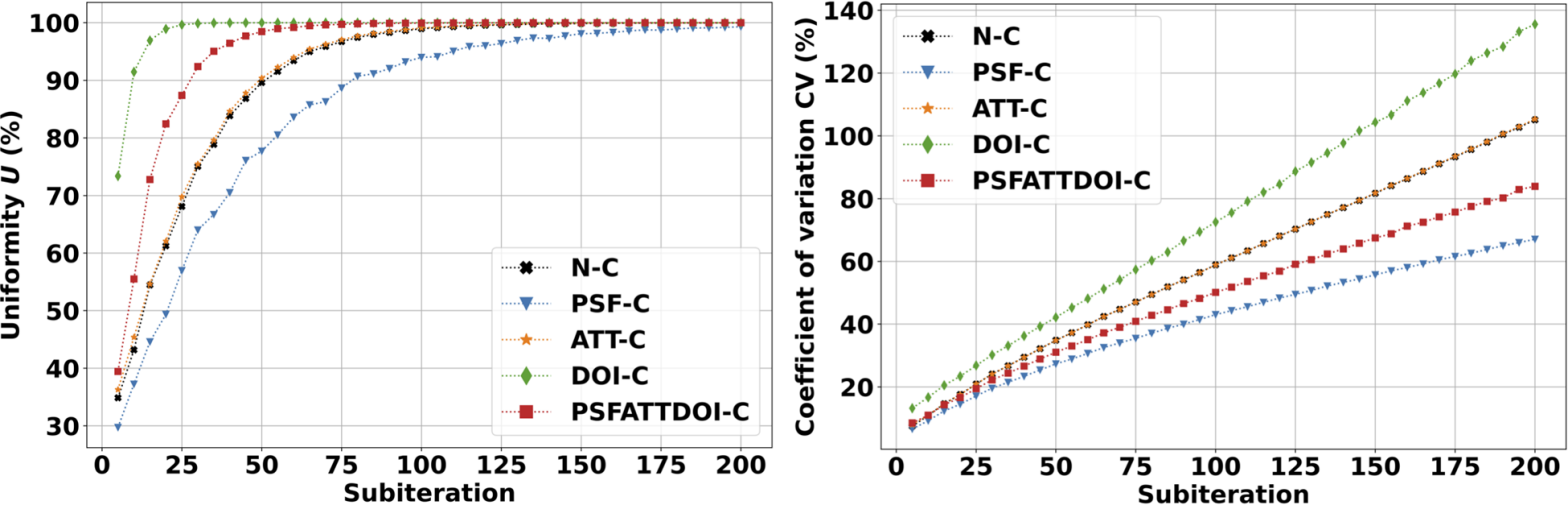
SPECT uniformity (**left**) and variability (**right**) in the volumetric cylinder. Images were reconstructed using the OSEM algorithm with seven subsets and various matrix corrections. As expected, uniformity and variability increased with increasing subiterations in the OSEM reconstruction. PSF correction improved uniformity and variability, while DOI correction degraded image quality due to a bug affecting voxels within a small angle from the pinhole axis.

**Figure 7:**
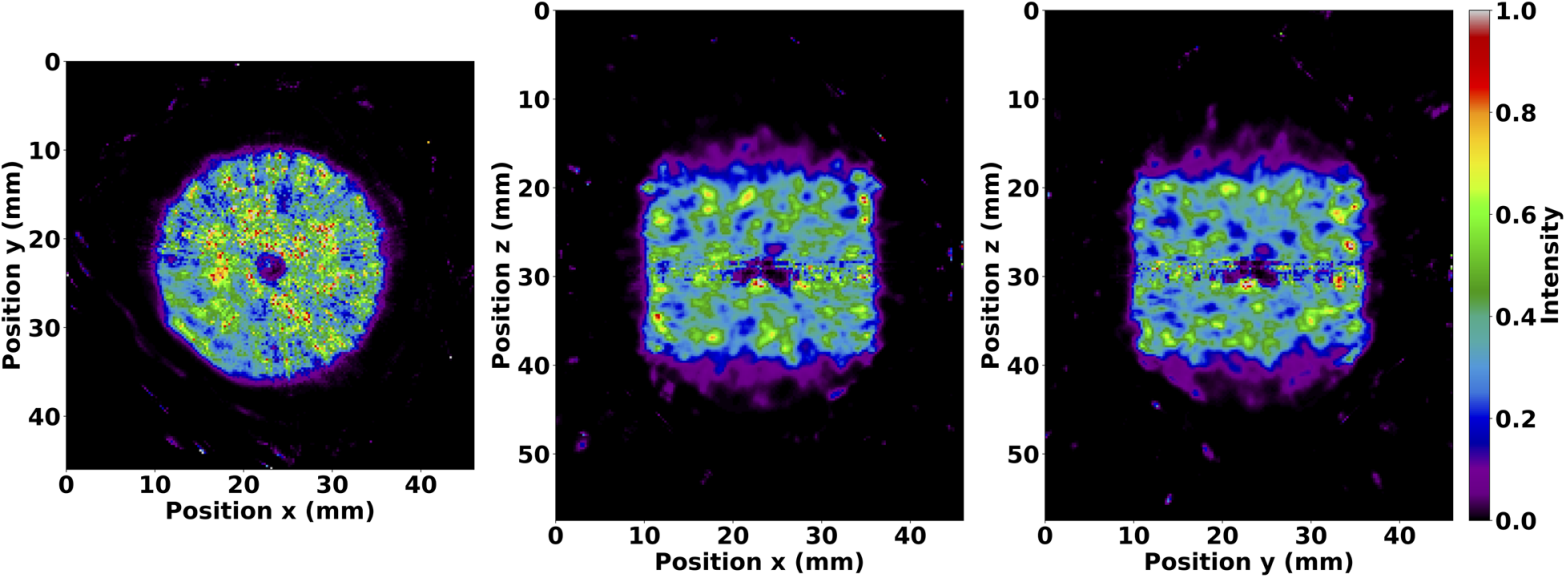
Illustration of the DOI bug shown in slices of the volumetric cylinder after 35 subiterations of the OSEM algorithm with DOI correction enabled. Image values were thresholded between 0 and 1. The effects from the bug are visible in the central transverse (**left**), coronal (**middle**), and sagittal (**right**) planes. The bug affects voxels within a small angle from the pinhole axis, as seen along the pinhole trajectory in a 270^∘^ counter-clockwise acquisition starting at 180^∘^. The transverse view shows the formation of a multi-armed cross or “star shot” artifact, and all views show the compounding effect at the isocenter due to the intersection of LORs affected by the bug.

Figure 6 presents uniformity and variability plots in the volumetric cylinder reconstructed with the OSEM algorithm. As expected, uniformity and variability worsened with increasing subiterations in OSEM reconstruction. PSF correction improved uniformity and variability amongst all independently applied matrix corrections, and attenuation correction provided no appreciable change in this preclinical application. DOI correction degraded uniformity and variability as illustrated in Figure 7 due to a bug affecting voxels within a small angle from the pinhole axis. This was reflected by a uniformity value that quickly reached 100% within five complete OSEM iterations and a CV with the largest slope and intercept compared to all other matrix calculations.

### 4.2. Qualitative assessment of reconstructed *in vivo* data

**Figure 8:**
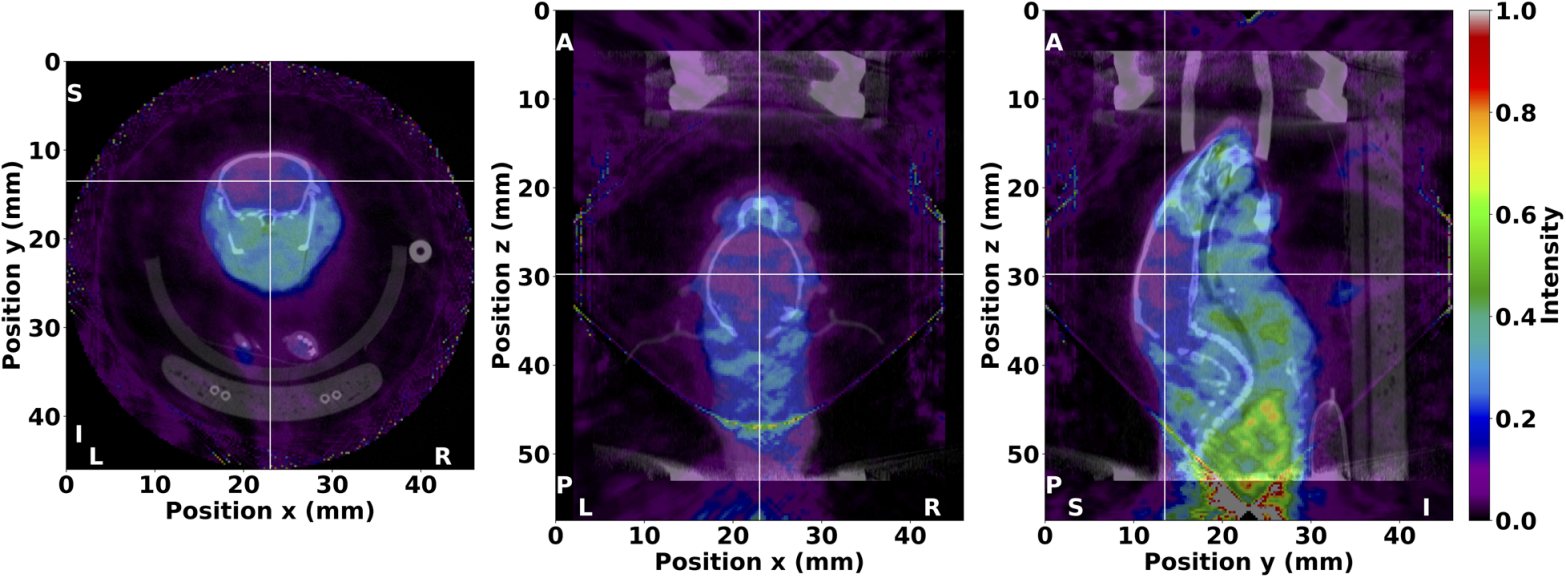
Fused SPECT/CT image of the *in vivo* mouse from the investigation of a potential ^123^I-labelled radiotracer for Alzheimer’s disease diagnosis. SPECT image values were thresholded between 0 and 1. The ^123^I distribution was clearly localised within the bounds of the body and various organs as illustrated in the transverse (**left**), coronal (**middle**), and sagittal (**right**) planes. Crosshairs are centered in the brain to denote the illustrated planes. The anatomical orientation is also shown with markers where L, left; R, right; A, anterior; P, posterior; S, superior; and I, inferior. The SPECT image was reconstructed with MLEM in nine iterations and no matrix corrections, and the μCT image was reconstructed with FBP and a ramp filter.

A fused SPECT/CT image of the *in vivo* mouse acquisition is shown in Figure 8, where the reconstructed radiotracer distribution was clearly localised within the bounds of the body and other organs. For example, the novel ^123^I-labelled tracer under investigation was observed in the olfactory bulb, eyes, salivary glands, and heart, with limited uptake in the brain. The conic bounds of the fully sampled FOV can also be seen in the fused SPECT/CT image, particularly in the posterior direction, from where the majority of γ-rays originated in this acquisition. Counts detected outside the fully sampled FOV are reconstructed with increased uncertainty, but images retain reliable localisation despite the extended distribution of radioactivity. Lastly, low-intensity background noise can be observed throughout the tomographic image. Overall, these results demonstrate that the PinholeSPECTUB projector is suitable for *in vivo* data, and tomographic images can be interpreted and analysed for further conclusions.

## 5. Discussion

The purpose of this study was to demonstrate and test the integration of a pinhole SPECT library in STIR using simulated and measured data. The aim was not to optimise reconstruction parameters for the γ-camera used in this study. Altogether, the SPH-SPECT images reconstructed with STIR showed appreciable image quality with radioactive source distributions true to their physical geometry. As discussed in the previous SPECTUB publication, reconstruction requires tuning of (sub)iterations for OSEM and MLEM algorithms or penalisation factors for OS-OSL-MRP and OS-SPS-QP algorithms based on the object size, activity, and background (6). In the present study, PSF correction improved image quality as seen in resolution, uniformity, and variability figures of merit. Although the inclusion of attenuation modelling improves system matrix accuracy, its present application in a preclinical setting shows minimal effects. In general, further improvements to image quality could be achieved with application-specific post-reconstruction image filtering (25).

Apart from the photon energy dependence in attenuation correction, PSF and DOI corrections include energy-specific factors for intrinsic resolution and crystal attenuation, respectively, to improve system matrix accuracy. When DOI correction is disabled, interactions in the scintillator are assumed to occur at half the crystal depth. An energy-dependent modification could apply a corresponding mean or median depth of interaction. This would affect reconstruction quality, and relatively small pinhole acceptance angles and degrees of parallax would be most accurate in the uncorrected case, while DOI correction would be required otherwise.

Unfortunately, DOI correction degrades image quality due to a bug affecting small angles from the pinhole axis. This results in a loss of counts where pinhole axes intersect rather than a distortion of the reconstructed radioactivity distribution, as illustrated in Figure 7. In our results, this caused a significant separation between maximum and minimum intensity values and resulted in uniformity quickly approaching 100%. When calculating CV, this caused an increased standard deviation and reduced mean, corresponding to greater variability in the cylindrical volume. However, the resolution calculated with DOI correction and reported in Figure 5 was negligibly affected since the resolution was reported as the average of all x and y FWHMs, and the axial line source was only affected in a fraction of the 3.5 mm-thick slice at the isocenter. More specifically, the mean relative standard error from all subiterations calculated with Eq. (2) was 9% for DOI-corrected matrices and 8% otherwise, which shows consistent resolution with minor variations throughout the tomographic FOV. Future work aims to correct the DOI bug.

The computational costs were comparable to STIR’s parallel- and converging-hole SPECTUB projector class. Matrices were chosen to be similar in size to those in the previous SPECTUB publication in (6), where projection and reconstruction matrix sizes were 1.1% and 3.2% larger, respectively. When storing the pinhole SPECT matrix in memory, computations required up to 3× more RAM and 4.7× more CPU time than the parallel-hole SPECT case, except for PSF correction, which required 1.7× less RAM and 1.2× less CPU time. When calculating the matrix per projection, memory requirements were nearly identical, and computations took up to 6× longer using the PinholeSPECTUB projector, except for PSF correction which required 1.3× less RAM and 1.1× less CPU time. The general increase in computation cost can be attributed to pinhole SPECT LORs that intersect more voxels at non-orthogonal angles than a parallel-hole collimator, and the differences in PSF correction can be attributed to the correction applied in detector space for the PinholeSPECTUB projector versus object space for the SPECTUB projector.

The integrated software is included in STIR release 5.1.0. Further extensions could expand the software to support non-circular orbits, improve energy dependence, model keel-edge or lofthole pinholes, enable parallel computing, and correct for knife-edge penetration. In addition, camera designs can be readily explored with the PinholeSPECTUB projector for single- and multi-pinhole collimators in terms of magnification, detector coverage, multiplexing, and pinhole geometry for optimal FOV, sensitivity, and detection efficiency without degrading spatial resolution (26). The Synergistic Image Reconstruction Framework (SIRF) (27) has also been extended to use these new STIR capabilities, allowing the use of SIRF’s advanced optimisation algorithms. Additional possibilities with the software include scatter correction, motion-compensated image reconstruction, synergistic image reconstruction, dynamic imaging, and multi-tracer protocols. Ongoing work aims to utilise the pinhole SPECT SIRF extension for multi-tracer protocols. The method under development requires multiple energy-dependent system matrices to simultaneously reconstruct distributions from a multi-radionuclide SPECT acquisition. However, this is currently not possible with STIR’s SPECT projectors, as the weight matrix is defined as a global variable that only allows for one unique matrix during reconstruction. Therefore, the next steps will replace any global variables with local ones.

## 6. Conclusions

Pinhole SPECT is becoming increasingly important in clinical and preclinical investigations of molecular imaging agents. We have demonstrated the pinhole SPECT modelling tool capabilities in the open-source STIR package. Tomographic image quality was evaluated qualitatively and quantitatively using several figures of merit and iterative reconstruction algorithms with and without system matrix corrections. Our results showed measurable and indicative image quality suitable for *in vivo* applications. This shows that STIR can be configured for complex pinhole SPECT scanner geometries and used with many reconstruction algorithms.

## Data availability statement

The raw data supporting the conclusions of this article will be made available by the authors, without undue reservation.

## Ethics statement

The animal study was reviewed and approved by Dalhousie University Committee for Laboratory Animals.

## Author’s contributions

MS wrote the ProjMatrixByBinPinholeSPECTUB class to integrate the prototype pinhole SPECT system matrix estimation software into STIR, with assistance from KT. MS also extended its integration to SIRF, performed data collection, image reconstruction, analysis, and wrote the manuscript. CF wrote the prototype pinhole SPECT system matrix estimation software. KE and BFH were involved in the development of the software. KB and KT contributed to study interpretation. All authors contributed to the article and approved the submitted version.

## Funding

MS was supported by a Nova Scotia Graduate Scholarship, and KB was supported by an NSERC grant (499115-2016). The prototype code was partly developed with support from the EC FP7 INSERT project (HEALTH-F5-2012-305311). STIR is partly maintained by the Collaborative Computational Project in Synergistic Reconstruction for Biomedical Imaging (CCP SyneRBI) through an EPSRC grant (EP/T026693/1). BFH acknowledges support from the NIHR UCLH Biomedical Research Centre.

## Appendix

This section presents part of a STIR parameter file for use with the PinholeSPECTUB projector. Sample detector and collimator files are also given below. Demonstrated parameters were configured for ^99*m*^Tc acquisitions using the Cubresa Spark. The STIR User’s Guide provides a detailed description of each parameter.

### Sample parameter file

projector pair type := Matrix

Projector Pair Using Matrix Parameters :=

Matrix type := Pinhole SPECT UB

Projection Matrix By Bin Pinhole SPECT UB Parameters:=

 maximum number of sigmas := 2.0 spatial resolution PSF := 0.01 subsampling factor PSF := 1

 detector file := detector.txt collimator file := collimator.txt

 ; PSF and DOI correction { Yes // No } psf correction := no doi correction := no

 ; Attenuation correction { Simple // Full // No } attenuation type := no attenuation map :=

 object radius (cm) := 2.3 mask file :=

 ; If no mask file is set, we can either compute it from attenuation map or object radius mask from attenuation map := 0

 keep all views in cache := 0

End Projection Matrix By Bin Pinhole SPECT UB Parameters:=

End Projector Pair Using Matrix Parameters :=

### Sample detector file

Number of rings: 1

#intrinsic PSF#

Sigma (cm): 0.0361

Crystal thickness (cm): 0.3

Crystal attenuation coefficient (cm -1): 4.407

#.........repeat for each ring.........#

Nangles: 91 ang0 (deg): 180. incr (deg): 3.0 z0 (cm): 0.

#..............until here..............#

### Sample collimator file

Model (cyl/pol): pol

Collimator radius (cm): 2.8

Wall thickness (cm): 1.

#holes#

Number of holes: 91 nh: ind x(cm) y(cm) z(cm) shape(rect-round) sizex(cm) sizez(cm) angx(deg) angz(deg) accx(deg) accz(deg) h1:  1  0. 0. 0.     round   0.1  0.1  0. 0.  45.  45.

⋮

h91: 91  0. 0. 0.    round   0.1  0.1  0.  0.  45.  45.
